# Extracellular vesicles secreted from bone metastatic renal cell carcinoma promote angiogenesis and endothelial gap formation in bone marrow in a time-dependent manner in a preclinical mouse model

**DOI:** 10.3389/fonc.2023.1139049

**Published:** 2023-03-23

**Authors:** Masashi Takeda, Hiromasa Sakamoto, Noboru Shibasaki, Tomohiro Fukui, Toshihiro Magaribuchi, Takayuki Sumiyoshi, Noriaki Utsunomiya, Atsuro Sawada, Takayuki Goto, Takashi Kobayashi, Koji Ueda, Toshinari Yamasaki, Osamu Ogawa, Shusuke Akamatsu

**Affiliations:** ^1^ Department of Urology, Kyoto University Graduate School of Medicine, Kyoto, Japan; ^2^ Project for Personalized Cancer Medicine, Cancer Precision Medicine Center, Japanese Foundation for Cancer Research, Tokyo, Japan

**Keywords:** extracellular vesicle (EV), renal cell carcinoma (RCC), bone metastasis (BM), angiogenesis, proteomics

## Abstract

**Introduction:**

Bone is a major metastatic site of renal cell carcinoma (RCC). Recently, it is well recognized that bone metastatic tumor cells remodel bone marrow vasculature. However, the precise mechanism underlying cell-cell communication between bone metastatic RCC and the cells in bone marrow remains unknown. Extracellular vesicles (EVs) reportedly play crucial roles in intercellular communication between metastatic tumor cells and bone marrow. Therefore, we conducted the current study to clarify the histological alteration in vascular endothelium in bone marrow induced by EVs secreted from bone metastatic RCC cells as well as association between angiogenesis in bone marrow and bone metastasis formation.

**Materials and methods:**

We established a bone metastatic RCC cell line (786-O BM) by in vivo selection and observed phenotypic changes in tissues when EVs were intravenously injected into immunodeficient mice. Proteomic analysis was performed to identify the protein cargo of EVs that could contribute to histological changes in bone. Tissue exudative EVs (Te-EVs) from cancer tissues of patients with bone metastatic RCC (BM-EV) and those with locally advanced disease (LA-EV) were compared for in vitro function and protein cargo.

**Results:**

Treatment of mice with EVs from 786-O BM promoted angiogenesis in the bone marrow in a time-dependent manner and increased the gaps of capillary endothelium. 786-O BM EVs also promoted tube formation *in vitro*. Proteomic analysis of EVs identified aminopeptidase N (APN) as a candidate protein that enhances angiogenesis. APN knockdown in 786-O BM resulted in reduced angiogenesis in vitro and in vivo. When parental 786-O cells were intracardially injected 12 weeks after treatment with786-O BM EVs, more bone metastasis developed compared to those treated with EVs from parental 786-O cells. In patient samples, BM-EVs contained higher APN compared to LA-EV. In addition, BM-EVs promoted tube formation in vitro compared to LA-EVs.

**Conclusion:**

EVs from bone metastatic RCC promote angiogenesis and gap formation in capillary endothelium in bone marrow in a time-dependent manner.

## Introduction

Kidney cancer accounts for 2.2% of cancer incidence and 1.8% of cancer-related deaths worldwide (1). Clear cell renal cell carcinoma (ccRCC) is the most common histopathological type of sporadic kidney cancer (2). About 15-30% of patients have metastases at initial presentation, and bone is one of the most common metastatic sites in ccRCC (3). Bone metastatic ccRCC is associated with poor prognosis due to poor response to contemporary systemic treatments, such as molecular targeted therapy and immune checkpoint inhibitors (4, 5). Moreover, bone metastasis causes skeletal-related events (SREs), such as severe pain, pathological fracture, hypercalcemia of malignancy, and spinal cord compression, resulting in diminished quality of life (6–8). Clinical benefit of current therapies targeting bone such as bisphosphonates and denosumab is limited to reduced SREs and prolonged time to SREs (9). Although earlier diagnosis of bone metastasis and intervention may lead to improved survival outcomes, the molecular basis underlying the early stages of bone metastasis remains largely unknown.

Angiogenesis plays crucial roles in tumor growth following metastatic colonization through delivering oxygen and nutrients (10, 11). It has already been reported that angiogenesis in bone marrow is promoted by growth factors such as vascular endothelial growth factor (VEGF) released by tumor cells colonizing in bone marrow (12). However, how cancer cells that have not metastasized yet to the bone marrow remotely affect angiogenesis in bone marrow for future metastasis is still understudied. As the tumor cell’s messenger, extracellular vesicle (EV) is gaining growing interest among researchers. EV is a nano-sized particle secreted from various cell types, containing biomolecules including proteins, lipids, DNA, and RNA, that are transferred to recipient cells with the potential to alter the phenotype of recipient cells (13, 14). EVs released from primary tumor reportedly contribute to cancer metastasis thorough promoting angiogenesis at future metastatic sites (15–17). In the majority of previous studies, EVs have been shown to alter the phenotypes of recipient cells immediately after uptake *in vitro* (18–20). However, considering the natural history of bone metastasis development, we hypothesized that *in vivo*, EVs secreted from bone metastatic ccRCC cells induce histological change in bone marrow capillaries over a substantial period of time. To test this hypothesis, we compared the functionality of EVs isolated from the culture supernatant of a bone metastatic ccRCC cell line (786-O BM) with those isolated from parental cells (786-O luc EV) and observed the vascular changes in bone marrow and metastasis formation over time *in vivo* after systemic injection of EVs in mice.

## Materials and methods

### Cell culture

The 786-O cell line (RRID: CVCL_1051) was purchased from the American Type Culture Collection (Rockville, MD, USA). Cells were cultured in Dulbecco’s modified Eagle’s medium (DMEM, Life Technologies, Netherlands). All cell lines were authenticated using short tandem repeat (STR) profiling within in the last three years. All experiments were performed with mycoplasma-free cells. Human umbilical vein endothelial cells (HUVECs, C-12203, RRID: CVCL_2959) were purchased from Promo Cell (Germany) and were cultured in endothelial cell growth medium (Promo Cell, Germany).

### Establishment of a bone metastatic ccRCC cell line

We generated stable luciferase expressing 786-O cells (786-O luc) using MSCV IRES Luciferase, which was gifted by Scott Lowe (Addgene #18760). We established a bone metastatic 786-O cell line using an *in vivo* selection method as described by Wang et al. with modification (21). The original protocol propagated RCC cells growing in bone from bone metastasis developed by tail vein injection of cells. In the present study, we directly injected 786-O cell line into tibial bone marrow to grow tumor in bone as described by Xie C et al. (22). In brief, 1×10^5^ cells in 10 μL phosphate-buffered saline (PBS) were injected into the tibial bone marrow cavity. Tumors in the hind limbs were monitored *via* bioluminescent imaging (BLI) using an *In Vivo* Imaging System (Lumina II, Caliper LS, USA). At 35 days post-injection, tumors detected *via* BLI were harvested. The muscles and skin were removed. Thereafter, the tumor was minced, dissociated with collagenase, and incubated at 37°C for 30 min. After centrifugation, the cells were resuspended in culture medium and seeded onto 6 cm dishes. We named this novel 786-O variant 786-O BM. To confirm enhanced bone metastatic capacity of 786-O BM, we injected 1×10^6^ 786-O BM cells into the left ventricle of nude mice and monitored bone metastasis formation *via* BLI (N=8 for each group).

### Cell proliferation assay

We seeded 1500 cells in 96-well plates in DMEM supplemented with 10% FBS. The cell proliferation assay was performed using the Cell Counting Kit-8 (Dojindo, Japan) according to the manufacturer’s instructions. Absorbance was measured at 450 nm and the experiments were performed in triplicate.

### EV isolation from cell culture

The cells were cultured in serum-free DMEM. After 24 h, the culture supernatant was sequentially centrifuged at 300 × *g* for 10 min and 2,000 × *g* for 10 min. After filtering through a 0.22 μm PVDF membrane (Merck Millipore, Germany), the supernatant was concentrated *via* ultrafiltration with Amicon Ultra-15 100 K (Merck Millipore). The concentrated supernatant was washed in PBS and purified by ultracentrifugation at 120,000 *× g* for 70 min (SW 60 Ti rotor, Beckman Coulter, USA). The pellet was washed in PBS again and subjected to another round of ultracentrifugation at 120,000 *× g* for 70 min. Finally, the EV pellet was resuspended in 200 μl of PBS. Protein content was measured using the Qubit Protein Assay Kit (Thermo Fisher Scientific, USA).

### EV isolation from patient tissue

We isolated tissue-exudative extracellular vesicles (Te-EVs) as described previously (23). Briefly, 10 mg of tissue samples collected from RCC patients were immersed in serum-free DMEM and incubated at 37°C for 3 h. The supernatant was sequentially centrifuged at 3,000 × *g* for 5 min and 12,000 × *g* for 30 min. The supernatant was pelleted by ultracentrifugation at 120,000 × *g* for 60 min (SW 60 Ti rotor, Beckman Coulter). The pellet EV was washed in PBS and ultracentrifuged at 120,000 × *g* for 60 min. This purification process was repeated once more, and the pellet was resuspended in 500 μl PBS.

### Transmission electron microscopy

EV samples (1μg) were placed on a carbon formvar copper grid for 10 min. After blocking with 4% BSA/PBS, the samples were incubated with primary antibody for 2 h. After washing in PBS, secondary antibodies conjugated with gold colloids (20 nm) were added to the samples and incubated for 2 h. The samples were then washed in PBS and fixed with 2% glutaraldehyde. Samples were incubated with 1% uranium acetate for 5 min, washed in DDW, and dried for 10 min. Observations were performed using the H-7650 transmission electron microscope (Hitachi, Japan).

Tibia of nude mice was also observed with TEM. After fixation with 4% paraformaldehyde and 2% glutaraldehyde in 0.1 M phosphate buffer, samples were decalcified with 10% EDTA for 72 h. Then, sample preparation was performed as previously described (24). In brief, samples were washed with 0.1 M PBS followed by post-fixation with 1% OsO_4_ and 0.1 M sucrose in 0.1 M phosphate buffer for 2 h. After dehydration, samples were embedded in epoxy resin. An area of interest, which was selected under a light scope was cut into ultra-thin 80-nm sections and placed on copper grids. Observation was performed with the H-7650 transmission electron microscope (Hitachi, Japan). We compared the number of endothelial gaps per capillary cross sections between those treated with 786-O luc EV and 786-O BM EV.

### Nano particle analysis

EV samples diluted with milliQ (1:50) were analyzed using a NanoSight NS300 (Quantum Design Japan). NTA 3.4 Build 3.4.003 was used for analysis.

### Protein extraction and western blotting analysis

Cells or EVs were lysed in RIPA buffer containing a protease inhibitor cocktail. Lysates were centrifuged at 15,000 rpm for 20 min. The supernatant was collected for immunoblotting analysis. Protein quantification was performed using the Pierce BCA Protein Assay Kit (Thermo Fisher Scientific, USA). Immunoblotting analysis was performed as described previously (24, 25). Anti-TSG101 (4A10), anti-CD63 (ab59479), and anti-CD13 antibodies (EPR4058) were purchased from Abcam. Anti-Vinculin antibody (E1E9V) was purchased from Cell Signaling Technology.

### Animal experiments

All experiments involving laboratory animals were performed in accordance with the Guidelines for Animal Experiments of Kyoto University (Permit Number 18240,19239). All animal experiments were conducted using 4- to 5- week-old female BALB/cAJcl nude (nu/nu) mice (CLEA, Tokyo, Japan). Mice were housed in individually ventilated cages with 6 mice per cage under specific-pathogen-free, controlled condition (constant room temperature, humidity, and 12h-light/dark cycle). Ad libitum access to food and filtered tap water was provided. During experiments, we conducted daily health and behavior checks on the mice. Mice were euthanized by CO2 inhalation when they were unable to eat or drink, had lost more than 20 percent of their initial body weight, exhibited a hunched posture, or had tumors larger than 20 mm. All procedures were performed under anesthesia with 2% isoflurane inhalation, and all efforts were made to minimize suffering. Following the experimental procedures, all animals were euthanized using 100% carbon dioxide, and animals’ deaths were confirmed by the absence of heartbeat and respiration. Thereafter, tumors were excised.

### EV pretreatment and histological examination *in vivo*

EVs secreted from ccRCC cell lines were collected using the above-mentioned method. EVs were intravenously injected *via* the tail vein or retro-orbital plexus at a dose of 10 μg protein per mouse every other day for two weeks. After the predefined intervals, the tibiae were harvested and subjected to histological analysis. We set four different intervals, 0,4,8, and 12-week (N=6 for each group). As a non-treatment control, we injected PBS instead of EVs. The total duration of this experiment for each group was 2,6,10, and 14 weeks. CD31 was used as an endothelial marker. The primary antibody used was mouse anti-CD31 antibody (CST #77699). Vascular density (VD) was defined as the percentage of vascular area per bone marrow. We calculated VD using NIH ImageJ software.

### Intracardiac injection after *in vivo* EV pretreatment

To examine whether angiogenesis induced by EVs is associated with bone metastasis development, we injected 1×10^6^ 786-O luc cells into the left ventricle of nude mice pretreated with 786-O luc EV or 786-O BM EVs 4 weeks or 12 weeks after the last EV injection (N=9 for each group). Control mice were treated with PBS before intracardiac injection following the same protocol (N=9). The total duration of this experiment was 24 weeks. Metastatic tumors were monitored *via* BLI using IVIS. Bone metastasis-free survival was estimated using the Kaplan-Meier curve. To verify the presence of human RCC colonization, metastatic tumors were subjected to immunohistochemical examination for AE1/AE3 (pan-cytokeratin marker). AE1/AE3 antibody (#M3515) was purchased from Dako (USA).

### Endothelial tube formation assay

We evaluated *in vitro* angiogenesis using the Endothelial Tube Formation Assay kit (Cell Biolabs, USA) following the manufacturer’s instructions. We seeded 2×10^4^ HUVECs onto a solidified ECM gel placed in a 96-well plate. The culture medium was replaced with growth factor-free DMEM. EVs were added to each well at a protein concentration of 20 μg/ml. Sixteen hours later, we measured the total tube length using NIH ImageJ software. HUVECs were used at passage 3 or 4. All experiments were performed in triplicate.

### Proteomic analysis

786-O luc or 786-O BM cells were cultured in DMEM supplemented with 10% exosome-depleted FBS for 24 h. Culture supernatants from 786-O luc or 786-O BM cells were subjected to sequential centrifugation at 300 × *g* for 10 min and 2,000 × *g* for 10 min. After filtration, EVs were isolated using MagCapture (FUJIFILM, Japan) following the manufacturer’s instructions. Proteomic analysis was performed by high-resolution LC/MS followed by data analysis, as previously described (26). The raw data are available at a public proteome database, Japan Proteome Standard Repository/Database (jPOST), ID JPST001971 (27).

Protein identification and label-free quantification were carried out on the Proteome Discoverer 2.2 software (Thermo Fisher Scientific, USA). SwissProt Human Database with Mascot (Matrix Science, USA) or Sequest HT (Thermo Fisher Scientific, USA) database search engines were used to search the LC/MS dataset for protein identification. The threshold for peptide identification was set at a false discovery rate (FDR) of <1%. When choosing candidate proteins for subsequent functional analysis, those with insufficient data (two or more missing values for each EV) were excluded.

### Establishment of *APN* knockdown cell line

To establish a cell line with stable knockdown of aminopeptidase N (APN)/CD13 in 786-O BM, we purchased short hairpin RNA (shRNA) lentiviral particles targeting APN (sc-29960-V) and control (sc-108080) from Santa Cruz Biotechnology (Santa Cruz, CA, USA), and carried out transduction following the manufacturer’s protocol. We named them shANPEP and shControl, respectively.

### Quantitative real-time PCR (qPCR)

Total RNA content was extracted from the shControl and shAPNEP cells. cDNA was synthesized from total extracted RNA using a ReverTra Ace qPCR RT Kit (Toyobo, Japan). qPCR was performed using SYBR Green PCR Master Mix (Thermo Fisher Scientific, USA). PCR reactions were performed in triplicate. The thermal cycling conditions were 95°C for 15 s, 60°C for 30 s, and 72°C for 30 s. The values were normalized to the levels of amplified glyceraldehyde-3-phosphate-dehydrogenase (*GAPDH*). The primer sequence for GAPDH was 5´-GAAGGTGAAGGTCGGAG

TC-3´ (sense) and 5´ -GAAGATGGTGATGGGATTTC-3´ (antisense). We used CD13 (h)-PR (#sc-29960-PR) as the primer for *APN* (Santa Cruz, USA).

### Human samples

Clinical specimens of ccRCC were obtained from patients with or without bone metastasis who underwent radical nephrectomy (RN) or biopsy at the Department of Urology, Kyoto University Hospital, with appropriate written informed consent under approval by Kyoto University’s Institutional Review Board (IRB approval number G52). We isolated Te-EVs from the supernatant of tissue samples collected from six patients with bone metastasis (BM-EV) and six patients with locally advanced disease (LA-EV). Patient characteristics are described in **Supplementary Table 1**. Of the six patients with bone metastasis, three patients also had lung metastasis and one patient had liver metastasis along with bone metastasis. However, we included these cases in our analysis because bone was the predominant site of metastasis in these cases with regard to tumor volume. For the control group, we included six non-metastatic locally advanced ccRCC patients whose pathological T stage was 3. Using Te-EVs from these patients, we evaluated angiogenesis induced by BM-EV or LA-EV *in vitro* by endothelial tube formation assay and measured the APN content of BM-EVs and LA-EVs by enzyme-linked immunosorbent assays (ELISAs).

### Enzyme-linked immunosorbent assay (ELISA)

Aminopeptidase N (APN/CD13) protein levels in Te-EVs were quantified using a CD13 ELISA kit (R&D Systems, Minneapolis, USA) according to the manufacturer’s protocol. Three independent wells were used for each sample. The total protein amount added to each well was set to 5 μg.

### Statistical analysis

Data are indicated as means ± standard error of measurement (SEM). The significance of differences between means was assessed using the Student’s *t*-test. Cell proliferation *in vitro*, tumor growth *in vivo* and vascular density were assessed using two-way repeated analysis of variance (ANOVA) with *post-hoc* Holm-Sidak test. The outcomes of the Kaplan-Meier curves were compared using the log rank test. Statistical significance was set at *P*<0.05. Statistical analyses were performed using GraphPad Prism version 6 (GraphPad Software, USA).

## Results

### Establishment and characterization of a bone metastatic ccRCC cell line

**Figure 1:**
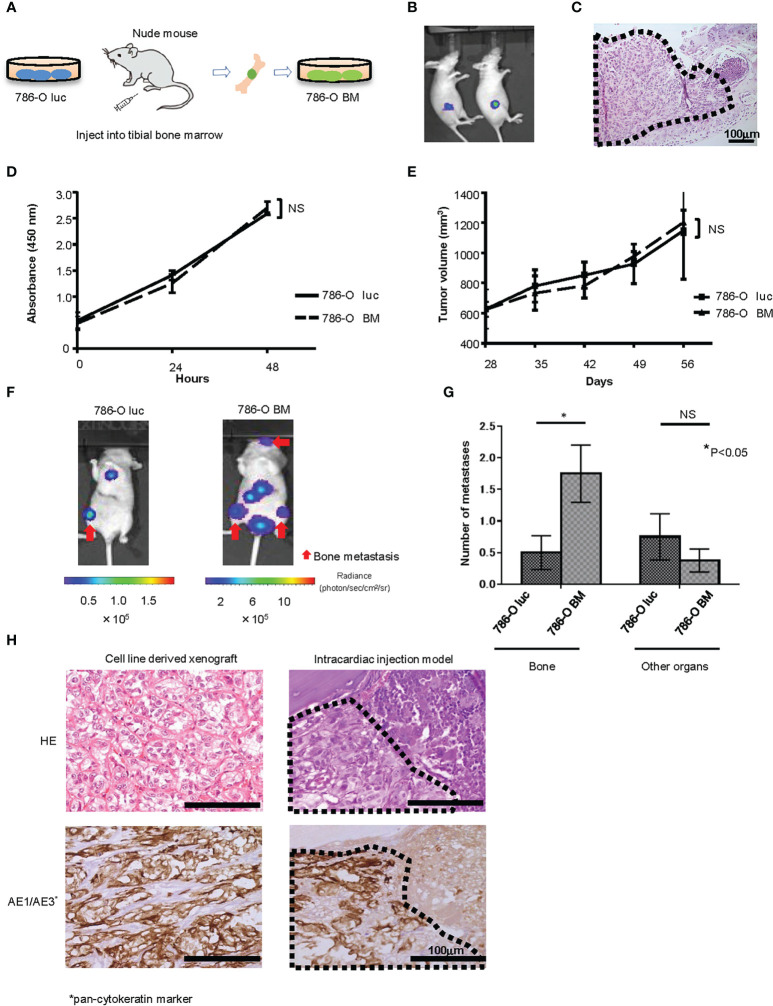
Establishment of a bone metastatic RCC cell line. **(A)** Schema of *in vivo* selection method. **(B)** Representative bioluminescent image of nude mice that underwent injection of 786-O luc cells into tibial bone marrow (Day 35). **(C)** H&E stain of developing bone tumor after 786-O luc cells were injected into tibial bone marrow. Black dashed lines surround metastatic tumor. Scale bar indicates 100 µm. **(D)** WST-8 assay of 786-O luc and 786-O BM cells. N=3. NS, not significant. Statistical significance was tested using two-way ANOVA with *post-hoc* Holm-Sidak test. **(E)** Subcutaneous xenograft assay of 786-O luc and 786-O BM cells. N=4. NS, not significant. Statistical significance was tested using ANOVA with *post-hoc* Holm-Sidak test. **(F)** Representative bioluminescent imaging of nude mice that received 786-O luc or 786-O BM cells *via* intracardiac injection. Red arrows indicate bone metastases, and non-arrowed lesions are metastasis other than bone metastasis. **(G)** Comparison of the number of bone metastasis and metastasis to other organs between mice injected with 786-O luc cells and 786-O BM cells. Statistical significance was tested using the Student’s t-test. N=8, *P<0.05. NS, not significant. **(H)** H&E stain and AE1/AE3 immunohistochemical stain of cell line-derived subcutaneous xenograft tumor of 786-O cells (left) and bone metastatic tumor in intracardiac injection model (right), both showing clear cell pathology. Black dashed lines surround metastatic tumor and AE1/AE3 positive cells are cancer cells. Scale bars indicate 100 µm.

We established a luciferase expressing 786-O (786-O luc) cell line and injected 786-O luc cells into the tibial bone marrow of nude mice to monitor tumor growth by BLI (**Figures 1A, B**). After confirming tumor growth in the bone marrow (**Figure 1C**), we named this cell line 786-O BM. 786-O BM cells displayed proliferation comparable to that of 786-O luc cells both *in vitro* and *in vivo* (**Figures 1D, E**). In contrast, 786-O BM cells showed an increase in bone metastasis *via* intracardiac injection in nude mice, while the number of metastases to other organs was comparable between the two cell lines. (**Figures 1F, G**). Similar to the cell line-derived subcutaneous xenograft tumor of 786-O BM, the bone metastatic tumor developed in an intracardiac injection model showed clear cell histology (**Figure 1H**).

### EV isolation from cell culture supernatant and characterization

We isolated EVs from the culture supernatant of 786-O luc and 786-O BM cells using the ultracentrifugation method. Isolated EVs were characterized using TEM, immunoblotting of EV marker proteins, and nanoparticle analysis. TEM analysis showed particles with a diameter of approximately 100 nm and spherical morphology in both 786-O luc and 786-O BM EVs (**Supplementary Figure 1A**). Immunoblotting confirmed the presence of CD63 and TSG101, widely known as EV markers, in both EVs (**Supplementary Figure 1B**). Nanoparticle Tracking Analysis showed an average particle diameter of 105.8 nm and 121.6 nm in 786-O luc and 786-O BM EVs, respectively (**Supplementary Figures 1C, D**). While particle concentration and protein content were higher in 786-O BM EVs than in 786-O luc EVs, the particle protein content ratio was similar in both groups (**Supplementary Figures 1E–G**).

### Histological changes induced by EV administration *in vivo*

**Figure 2:**
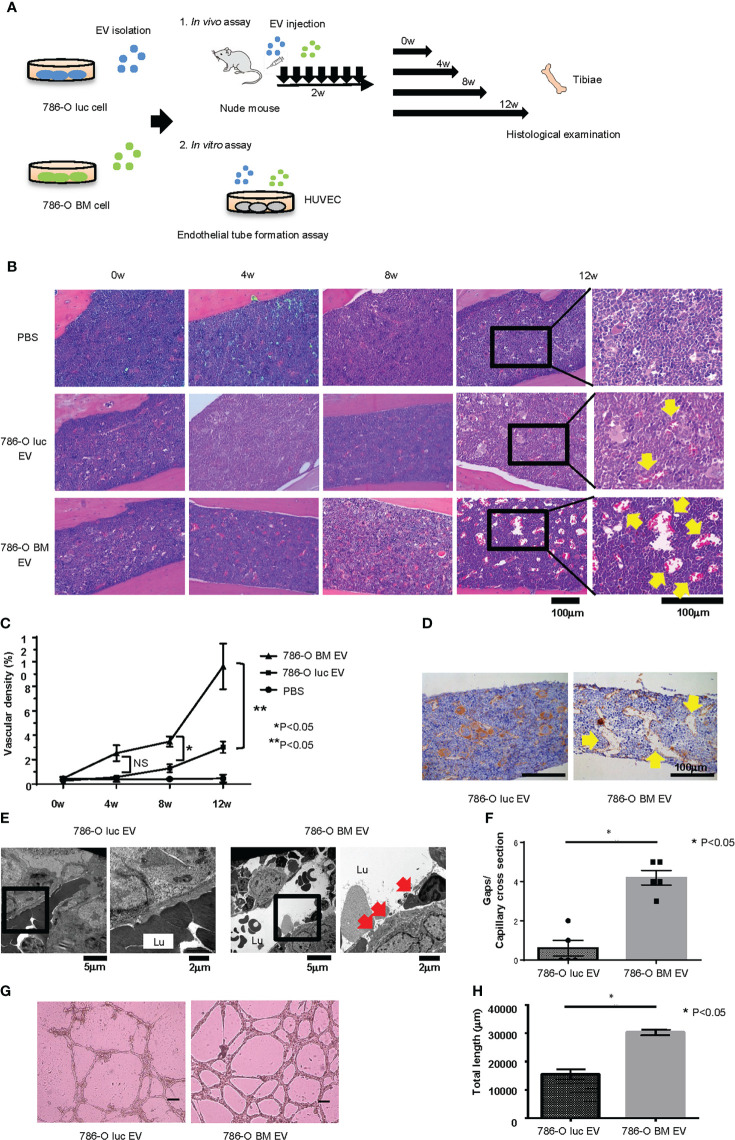
Histological changes induced by EV administration *in vivo* and endothelial tube formation assay *in vitro*. **(A)** Schema showing overview of the study exploring phenotypic changes after EV administration *in vivo* and *in vitro*. **(B)** H&E stain of tibial bone of nude mice treated with 786-O luc or 786-O BM EVs. Tibiae were harvested at 0, 4, 8, or 12 weeks after the last EV injection. Scale bars indicate 100 µm. Yellow arrows indicate dilated blood vessels. **(C)** Comparison of vascular density (VD) of tibial bone marrow between nude mice pretreated with 786-O luc and 786-O BM EVs. Intervals between EV treatment and harvest were set at 0, 4, 8 or 12 weeks. Data represent means ± SEM. Statistical significance was tested using the two-way ANOVA with *post-hoc* Holm-Sidak test. N=6 for those treated with 786-O luc EV or 786-O BM EV, N=3 for PBS control. *P<0.05. NS, not significant. **(D)** CD31 (endothelial marker) immunohistochemistry of tibial bone of nude mice treated with 786-O luc or 786-O BM EVs. Tibiae were harvested 12 weeks after the last EV injection. Scale bars indicate 100 µm. Yellow arrows indicate dilated blood vessels. **(E)** Transmission electron microscopy (TEM) images. Lu: Capillary lumen. Red arrows indicate endothelial gaps. Notice that in the bone marrow of 786-O luc EV treated mice, endothelial cells are tightly aligned to surround the capillary lumen whereas there are loose endothelial gaps in the 786-O BM EV treated mice. **(F)** Comparison of number of gaps per capillary cross section. Data represent mean ± SEM. Statistical significance was tested using the Student’s t-test. N=5, *P<0.05. **(G)** Representative imaging of endothelial tube formation assay results. Scale bars indicate 100 µm. **(H)** Comparison of total tube length calculated using Image J software. Data represent means ± SEM. Statistical significance was tested using the Student’s t-test. N=5, *P<0.05.

To examine the histological changes after treatment with 786-O luc and 786-O BM EVs *in vivo*, we intravenously injected 786-O luc or 786-O BM EVs into nude mice at a dose of 10 μg every other day for two weeks and compared the histological findings of tibiae after a set of intervals (**Figure 2A**). Histological examination revealed an increase in dilated blood vessels in the tibial bone marrow of 786-O luc EV-injected mice and 786-O BM EV-injected nude mice compared with PBS control in a time-dependent manner, indicating that EV injection results in enhanced angiogenesis and that the effect of EV injection becomes more apparent over time (**Figures 2B, C**). Furthermore, 786-O BM EV-injected nude mice showed significantly higher vascular density at 8-weeks and 12-weeks timepoints after EV treatment compared with 786-O luc EV-injected mice. Immunohistochemically, the endothelial cells were positive for CD31 (**Figure 2D**). In addition, we performed histological examination of the liver, lung, spleen, and kidney to examine the long-term effects of EV treatment on these organs. We found slight sinusoidal dilation in the liver of those treated with 786-O BM EVs compared to those treated with 786-O luc EVs. Conversely, there was no significant difference in the histological findings in the lung, spleen, and kidney (**Supplementary Figure 2**). Next, to observe microvasculature in bone marrow, we performed TEM analysis. The result showed increased gaps in capillary endothelium of bone marrow in those treated with 786-O BM EV (**Figures 2E, F**).

### *In vitro* endothelial tube formation assay

To test whether 786-O BM EVs induce angiogenesis *in vitro*, we performed an endothelial tube formation assay. HUVECs were cultured in medium containing 786-O luc or 786-O BM EVs for 16 hours, and the total tube length was measured using NIH ImageJ software. HUVECs treated with 786-O BM EVs showed an extended total tube length (**Figures 2G, H**).

### Proteomic analysis

**Figure 3:**
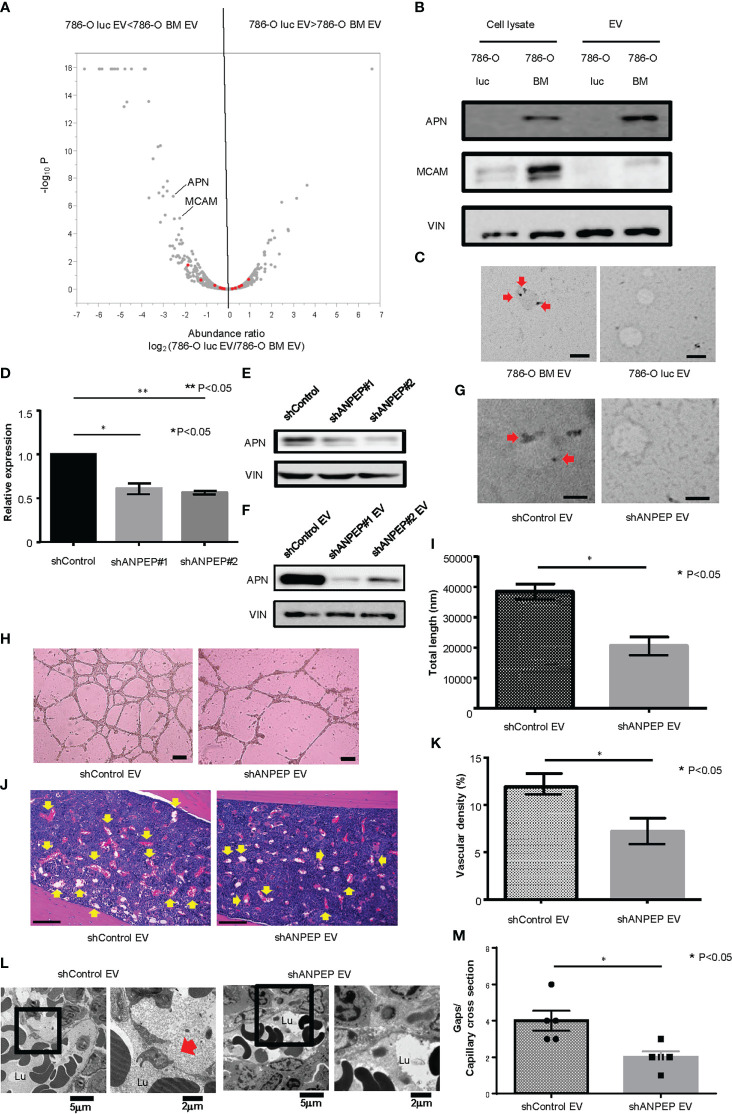
Proteomic analysis and establishment of an APN knockdown cell line. **(A)** Volcano plot showing a distinct protein profile of 786-O luc and 786-O BM EVs. Red dots indicate EV marker proteins (e.g., tetraspanins). **(B)** Western blotting of APN and MCAM. APN, Aminopeptidase N, MCAM, Melanoma Cell Adhesion Molecule, VIN, Vinculin. **(C)** Immunoelectron microscopic imaging of 786-O luc and 786-O BM EVs. Anti-APN primary antibody was used. The secondary antibody was labeled with 20 nm gold colloids (red arrows). Scale bars indicate 100 nm. **(D)** mRNA expression levels of APN in shControl and shANPEP cells measured using qRCR. Data represent means ± SEM. Statistical significance was tested using the Student’s t-test. N=3, *P<0.05, **P<0.05. **(E)** Western blotting results showing decreased APN protein in shANPEP cells. **(F)** Western blotting results showing decreased APN protein in shANPEP EV. **(G)** Immunoelectron microscopic imaging of shControl and shANPEP EVs. Anti-APN primary antibody was used. The secondary antibody was labeled with 20 nm gold colloids (red arrow). Scale bars indicate 100 nm. **(H)** Representative imaging of endothelial tube formation assay results. Scale bars indicate 100 µm. **(I)** Comparison of total tube length calculated using ImageJ software. Data represent means ± SEM. Statistical significance was tested using the Student’s t-test. N=6, *P<0.05. **(J)** H&E stain of tibial bone of nude mice pretreated with shControl or shANPEP EVs. Scale bars indicate 100 µm. Yellow arrows indicate dilated blood vessels. **(K)** Comparison of Vascular density of tibial bone marrow between nude mice pretreated with shControl and shANPEP EVs. Data represent means ± SEM. Statistical significance was tested using the Student’s t-test. N=6, *P<0.05. **(L)** Transmission electron microscopy (TEM) images. Lu: Capillary lumen. Red arrows indicate endothelial gaps. **(M)** Comparison of number of gaps per capillary cross section. Data represent mean ± SEM. Statistical significance was tested using the Student’s t-test. N=5, *P<0.05.

To explore the protein contents of EVs responsible for enhanced angiogenesis and increased gaps in bone marrow capillary, we performed a proteomic analysis of 786-O luc and 786-O BM EVs using liquid chromatography (LC)/mass spectrometry (MS). Data analysis identified a total of 1336 proteins, among which 38 and 167 proteins were significantly enriched in 786-O luc and 786-O BM EVs, respectively (**Figure 3A**). Recently, an increasing number of researchers have reported that membranous proteins of EVs play crucial roles in intercellular communication between cancer cells and the tumor microenvironment (28, 29). Therefore, we focused on membranous proteins among the top 20 proteins enriched in 786-O BM EVs as compared with 786-O luc EVs with a statistically significant difference (**Supplementary Table 2**). We detected five membranous proteins: gap junction alpha 1 (also known as Connexin43), ADP/ATP translocase 2, vesicle-associated membrane protein 3 (VAMP3), aminopeptidase N (also known as CD13), and cell surface glycoprotein MUC18 (also known as MCAM). Among these five proteins, we selected aminopeptidase N (APN) and MCAM, which showed abundant unique peptides and higher Mascot and Sequest HT scores as candidate targets (**Supplementary Table 3**). Both proteins were enriched in cell lysates of 786-O BM cells compared with 786-O luc cells. However, only APN was enriched in 786-O BM EVs compared with 786-O BM cells (**Figure 3B**). Moreover, immune electron microscopy revealed an increase in APN located on the 786-O BM EV membrane (**Figure 3C**). Therefore, we focused on APN as a target protein for further functional analyses.

### Establishment of an APN knockdown cell line and functional analysis

To test the function of APN in angiogenesis, we knocked down APN in 786-O BM cells using an shRNA system (shANPEP). shANPEP cells showed reduced APN expression at both the mRNA and protein levels (**Figures 3D, E**). APN was also reduced in EV secreted from shANPEP cells (**Figure 3F**). Immunoelectron microscopic analysis revealed a decrease in APN localization at the membrane of shANPEP EVs compared with shControl EVs (**Figure 3G**). HUVECs cultured in medium with shANPEP EVs showed decreased total tube length in the tube formation assay compared to those treated with shControl EVs (**Figures 3H, I**). Histological examination of the tibial bone marrow of nude mice pretreated with shANPEP EVs revealed reduced angiogenesis compared to those pretreated with shControl EVs, albeit to a lesser extent than the difference observed between treatment by 786-O luc EV and 786-O BM EV (**Figures 3J, K**). To examine impact of APN on microvasculature in bone marrow, TEM analysis was performed. The result showed a decreased endothelial gap formation in bone marrow of nude mice treated with shANPEP EV compared to those treated with shControl EV (**Figures 3L, M**), suggesting that EV-APN could promote angiogenesis and endothelial gap formation in bone marrow.

### Intracardiac injection of 786-O luc cells into EV-treated mice

**Figure 4:**
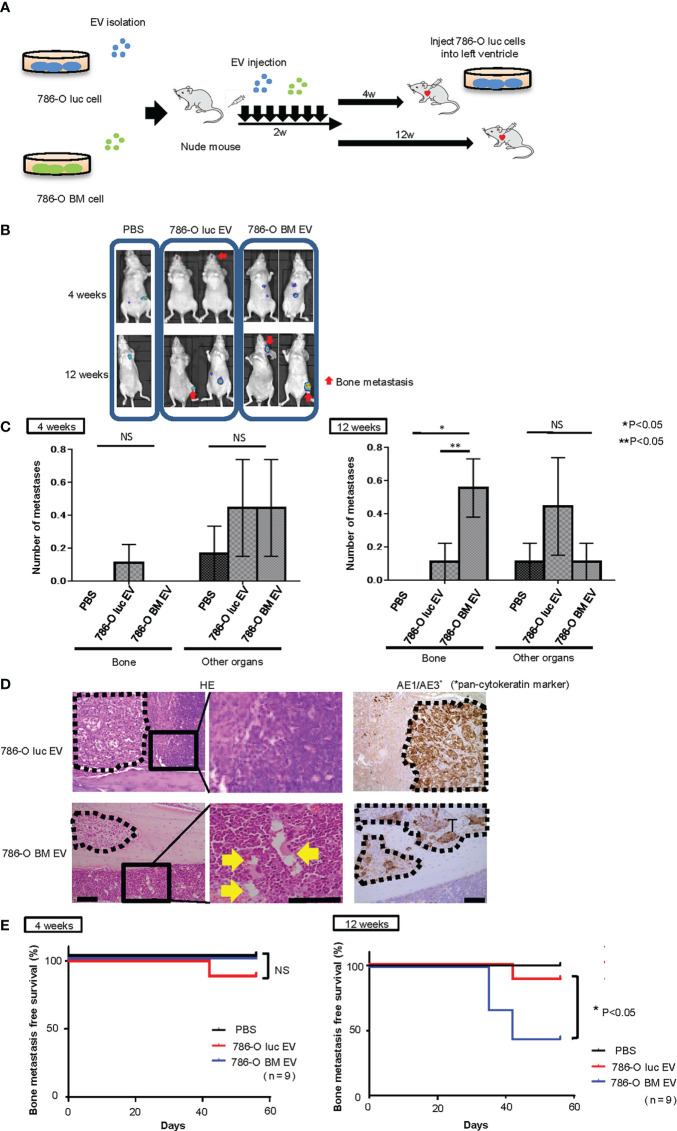
Intracardiac injection of cancer cells into nude mice pretreated with EVs. **(A)** Schema showing study protocol. **(B)** Representative bioluminescent imaging of nude mice pretreated with PBS,786-O luc EVs or 786-O BM EVs that developed metastases after intracardiac injection of 786-O luc cells. Intracardiac injection was performed 4 weeks or 12 weeks after the last EV injection. Red arrows indicate bone metastases, and non-arrowed lesions are metastasis other than bone metastasis. **(C)** Comparison of the number of bone metastasis and metastasis to other organs between mice treated with PBS, 786-O luc EVs or 786-O BM EVs. Intracardiac injection of 786-O luc cells were performed 4 weeks or 12 weeks after last pretreatment. Data represent means ± SEM. Statistical analysis was performed two-way ANOVA with *post-hoc* Holm-Sidak test. N=9 for each group. *P, **P <0.05. NS, not significant. **(D)** H&E stain and immunohistochemical stain of AE1/AE3 of tibial bone metastasis. Black dashed lines surround metastatic tumor. Yellow arrows indicate dilated blood vessels. Scale bars indicate 100 µm. **(E)** Kaplan-Meier curve comparing bone metastasis free survival of nude mice pretreated with PBS, 786-O luc EVs or 786-O BM EVs. Intracardiac injection was performed 4 weeks or 12 weeks after the last EV injection. Statistical significance was tested using the log rank test. N=9 for each group, *P<0.05. NS, not significant.

Next, to test whether angiogenesis and microstructural alteration in bone marrow endothelium induced by pretreatment with 786-O BM EVs is associated with bone metastasis formation *in vivo*, we monitored bone metastasis with BLI in nude mice injected with 786-O luc cells into the left ventricle 4 or 12 weeks after the last pretreatment with EVs (**Figure 4A**). We found increased bone metastasis in nude mice pretreated with 786-O BM EVs compared to those pretreated with PBS control or 786-O luc EVs in the 12-weeks pretreatment interval arm (**Figures 4B, C**). Among the 12-weeks pretreatment interval group, five out of nine of those pretreated with 786-O BM EVs developed bone metastasis, while bone metastasis was observed in only one mouse among those pretreated with 786-O luc EVs. In total, 786-O BM EV-treated mice developed five bone metastases (83%) and one subcutaneous metastasis (17%), while 786-O luc EV-treated mice developed one bone metastasis (20%), one lung metastasis (20%) and three subcutaneous metastases (60%), indicating bone dominant metastasis in the former. A significant difference was observed in the histological findings of bone metastatic tumors in the tibia between the two groups. Bone metastatic tumor cells in nude mice treated with 786-O BM EVs colonized the bone cortex with dilated blood vessels in the adjacent bone marrow. In contrast, tumor cells colonized the bone marrow in those treated with 786-O luc EVs (**Figure 4D**). We analyzed the bone metastasis-free survival of nude mice injected with 786-O luc or 786-O BM EVs using the Kaplan-Meier method. The results showed a statistically significant difference in bone metastasis-free survival between the two groups (**Figure 4E**). To evaluate bone metastasis formation at an earlier time point after treatment with EVs, we also conducted a similar experiment with EV pretreatment for four weeks. Interestingly, none of the nine mice with intracardiac injection of cancer cells four weeks after treatment with 786-O BM EVs developed bone metastasis (**Figures 4B, C, E**).These data clearly show that angiogenesis induced by 786-O BM EV over a long period of time strongly correlates with bone metastasis formation in our model. Next, to test whether EV-APN could directly contribute to bone metastasis development, we conducted intracardiac injection to nude mice pretreated with shANPEP EV or shControl EV 12 weeks after the last EV injection. Among those pretreated with shANPEP EV, four out of nine mice developed bone metastasis while five out of nine mice pretreated with shControl EV developed bone metastasis. The lack of statistical significance between the two groups in terms of bone metastasis-free survival suggests that EV-APN alone is not sufficient to facilitate bone metastasis development (**Supplementary Figures 3A, B**).

### Analysis of Te-EVs derived from tissue of RCC patients

**Figure 5:**
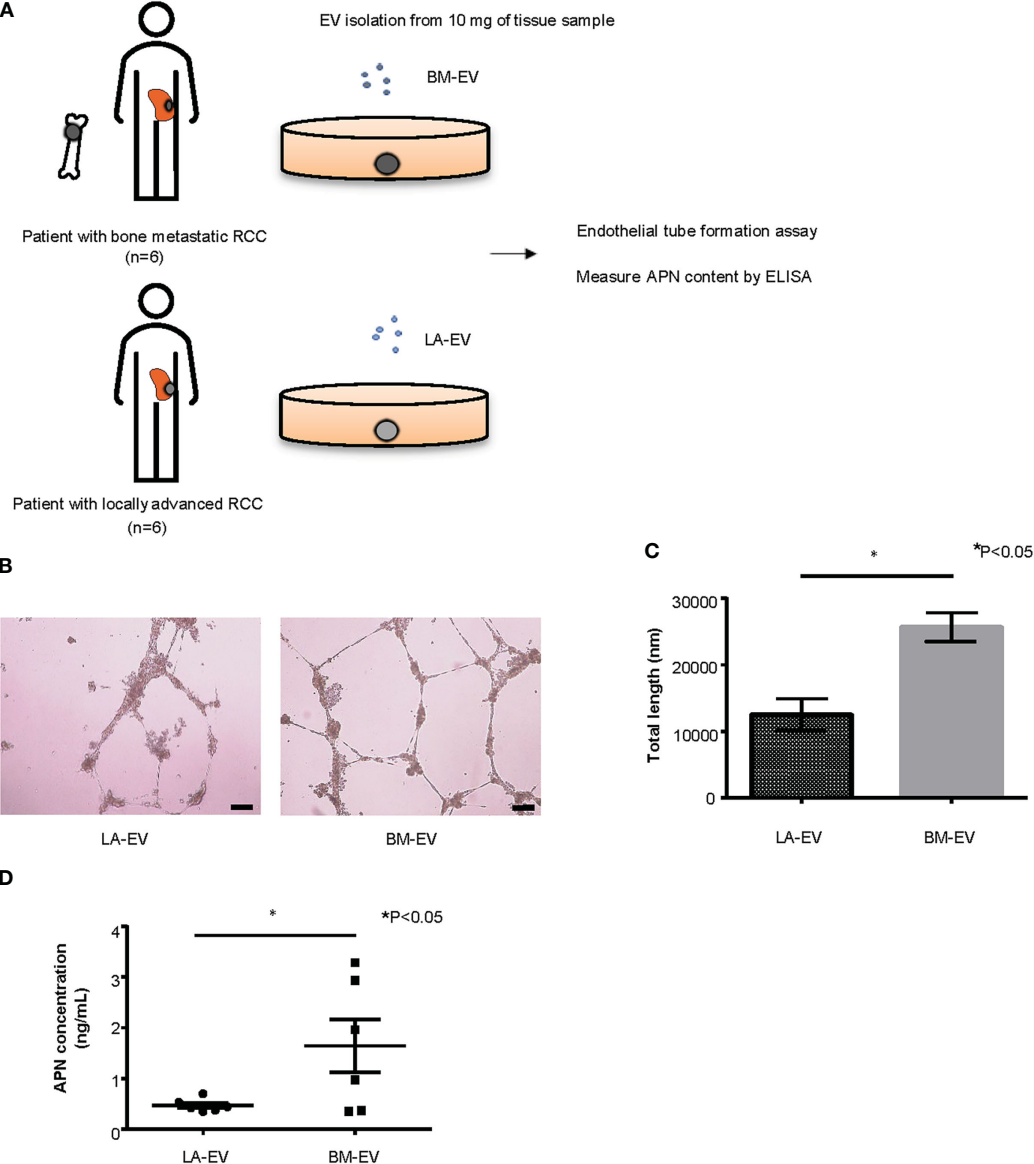
Analysis of Te-EVs from patients with metastatic or locally advanced ccRCC. **(A)** A schema showing an overview of the experiment. We extracted Te-EV from cancer tissues of patients with bone metastatic ccRCC (BM-EV) and those with locally advanced disease (LA-EV).Then we compared how they affect angiogenesis by endothelial tube formation assay. APN content in Te-EV was measured by ELISA and compared between the two groups. N=6 for each group. **(B)** Representative imaging of endothelial tube formation assay results. Scale bars indicate 100 µm. **(C)** Comparison of total tube length calculated using ImageJ software. Data represent means ± SEM. Statistical significance was tested using the Student’s t-test. N=6, *P<0.05. **(D)** APN levels determined using ELISA. Data represent means ± SEM. Statistical significance was tested using the Student’s t-test. N=6, *P<0.05.

Next, we examined the functionality of tissue exudative EVs (Te-EVs) from patients with bone metastatic and non-bone metastatic RCC and evaluated the expression of APN in Te-EV. For this purpose, Te-EVs were collected from primary tumors of six patients with bone metastasis (BM-EV) and six patients with locally advanced RCC (LA-EV) with no bone metastasis. All included patients were histologically diagnosed with clear cell RCC. There were no statistically significant differences in patient characteristics, such as age, sex, clinical T stage, and pathological T stage (**Figure 5A**, **Supplementary Table 1**).

First, to test whether Te-EVs secreted from ccRCC patients with bone metastasis could induce angiogenesis, we observed tube formation of HUVECs cultured in media with BM-EVs or LA-EVs. The results showed significantly increased tube formation in HUVECs treated with BM-EVs compared to those treated with LA-EVs (**Figures 5B, C**). Next, we measured APN levels in EVs using ELISA. Remarkably, three out of six patients with bone metastasis showed elevated levels of APN in Te-EV, while there was no elevation of APN in Te-EVs collected from patients without bone metastasis. The mean APN content in Te-EVs differed significantly between the two groups (**Figure 5D**). These data suggest that EVs secreted from RCC patients with bone metastasis have a higher angiogenic capacity with increased APN content.

## Discussion

In the present study, we have revealed that bone metastatic RCC has the capacity to promote angiogenesis and endothelial gap formation in bone marrow prior to DTC colonization and that these histological changes in bone marrow are mediated by EVs. Although a recent study has shown that bone metastatic solid tumor cells have the property to affect bone marrow vasculature following DTC colonization (30), whether tumor cells can change the vasculature in bone marrow prior to colonization remained elusive. In the past, many investigators have tried to show that EVs secreted from metastatic cancer cells promote metastasis through angiogenesis by inoculating cancer cells with EVs, or immediately after treatment with EVs. For example, Kosaka et al. demonstrated that EVs secreted from metastatic breast cancer cells induced angiogenesis through the function of miR-210, affecting endothelial cells (15). Zeng et al. showed that metastatic colorectal cancer-derived EVs facilitated angiogenesis at metastatic sites through the function of miR-25-3p (16). Grange et al. demonstrated that CD105-positive renal cancer cells secrete EVs that induce activated angiogenesis, leading to premetastatic niche formation in the lung, and increased lung metastasis after intravenous injection (17). However, no previous study has examined the effect of EVs on the bone microenvironment after a prolonged time. Considering the natural history of RCC, bone metastasis is always preceded by the growth of primary tumors. According to Santini et al., the median time to bone metastasis was 25 months in patients without bone metastasis at initial diagnosis (31). In the present study, we examined how EVs affect the bone microenvironment in the long term by examining histological changes of bone marrow and bone metastasis formation four and 12 weeks after treatment with EVs. The results of our study showed increased angiogenesis in the tibial bone marrow of nude mice treated with EVs collected from bone metastatic cancer cells beginning at four weeks after the last EV injection; however, the dilated vessels further increased at 8 and 12 weeks after treatment in a time dependent manner. Importantly, mice pretreated with 786-O BM EVs for 12 weeks showed increased bone metastasis among total metastasis after intracardiac injection of 786-O cancer cells compared to those that were pretreated for four weeks, suggesting a correlation between angiogenesis in bone marrow and bone metastasis formation. In addition to angiogenesis, treatment with EVs secreted from bone metastatic RCC was associated with increased endothelial gaps in bone marrow. A recent review described that tumor blood vessels undergo continuous growth and remodeling over an extended period, resulting in increased gaps in endothelium and enhanced vascular permeability (32). Formation of endothelial gap in bone marrow over 12 weeks after EV treatment suggests that EVs promote vascular microenvironment typical for tumor blood vessels.

Among the top 20 proteins enriched in 786-O BM EVs compared to 786-O luc EVs, we selected five membranous protein, Connexin 43, ADP/ATP translocase 2, VAMP 3, MCAM and APN. The roles of APN, MCAM, and ADP/ATP translocase 2 in EVs have not been fully investigated, although Connexin 43 and VAMP 3 are widely recognized as EV components (33, 34). Therefore, considering the novelty as EV cargo, abundant peptide scores and enrichment in EV, we finally focused on APN, a membrane-bound metalloproteinase-degrading extracellular matrix protein that contributes to cancer invasion and metastasis (35, 36). The soluble form of APN reportedly facilitates angiogenesis and inflammation (37). In our study, knockdown of *APN* reduced APN expression in EVs and resulted in impaired angiogenesis both *in vitro* and *in vivo*. Moreover, endothelial gap formation in bone marrow was also reduced. ELISA results showed higher APN levels in Te-EVs isolated from ccRCC patients with bone metastasis compared with those isolated from patients without bone metastasis. Hensbergen et al. demonstrated that the soluble form of APN was elevated in the plasma and effusions of cancer patients and correlated with tumor burden (38). Our findings suggest that EV-APN contributes to increased angiogenesis and vascular remodeling in the bone marrow of bone metastatic ccRCC. However, *in vivo*, treatment with shANPEP EVs was not sufficient to decrease bone metastasis formation, suggesting that EV-APN alone is not sufficient to facilitate premetastatic niche formation. Recent studies revealed that EV-APN and soluble APN may have different mechanisms of action, with EV-APN stimulating NFkb signaling by binding to the TLR4 receptor while soluble APN binds to the G protein coupled receptor (GPCR) (36, 39). Therefore, further research is required to fully understand the molecular basis underlying the angiogenesis facilitated by EV-APN. Our study had several limitations. First, we only conducted a proteomic analysis of EVs. Since EVs contain other biomolecules, such as microRNA, DNA, and lipids, enhanced angiogenesis and bone metastasis formation induced by EVs from the bone metastatic RCC cells are also likely affected by biomolecules other than APN. In addition, the gradual promotion of angiogenesis and endothelial gap formation after EV injection suggest the presence of complex signaling networks underlying premetastatic niche formation in bone that are partially modulated by EVs. Considering that endothelial gap formation causes decreased endothelial barrier function and increased vascular permeability, leading to extravasation of plasma and proteins into stroma, it is also likely that vascular remodeling caused by EVs enhanced additional factors to modulate stromal remodeling in bone marrow (30, 40). Second, due to only partial angiogenesis inhibition by APN knockdown, we were unable to demonstrate a direct relationship between bone marrow vascular remodeling and increased bone metastases. Future research should be conducted to clarify whether angiogenesis in bone marrow directly promotes bone metastasis. Third, we did not examine the detailed architecture of bone metastasis that formed in mice after treatment with EVs. Our data showed histological differences in bone metastatic tumors that developed by intracardiac injection between nude mice pretreated with 786-O BM EVs and those pretreated with 786-O luc EVs. These data imply that 786-O BM EVs may induce specific histological alterations in bone. Some reports have demonstrated that EVs affect bone by inducing osteoclast differentiation and activation, leading to increased osteolytic metastasis (41–43). Further research is required to explore the mechanism by which 786-O BM EVs alter the bone architecture and to identify key biomolecules in EV cargo playing crucial roles in osteoclast interaction, which could cause tumor premetastatic niche formation in bone. Finally, our *in vivo* experiments were conducted using immunocompromised mice. It has been reported that EVs carry biomolecules that can affect immune responses (44). Therefore, the functional consequences of EVs in human bone metastatic RCC may be more complex than that observed in our model. Nonetheless, the present study showed, for the first time, the long-term effect of EVs on the vascularity in bone marrow, which would enhance our understanding of the function of EVs.

## Data availability statement

The datasets presented in this study can be found in online repositories. The names of the repository/repositories and accession number(s) can be found in the article/**Supplementary Material**.

## Ethics statement

The studies involving human participants were reviewed and approved by Kyoto University’s Institutional Review Board. The patients/participants provided their written informed consent to participate in this study. The animal study was reviewed and approved by Kyoto University Institutional Animal Care and Use Committee.

## Author contributions

MT performed *in vivo* and *in vitro* experiments and drafted the manuscript. HS has established 786-O BM cell line. NS and NU have established the intracardiac injection protocol. TF and TM contributed to *in vivo* experiments. TS, TG, TY, and TK contributed to experimental design, interpretation of data and editing of the manuscript. AS collected clinical samples. KU performed proteomic analysis. OO acquired the research funding and supervised the work. SA contributed to research conception, obtained funding, drafted the manuscript, and supervised the work. All authors contributed to the article and approved the submitted version.
