# Supplementary material for: Extracellular vesicles secreted from bone metastatic renal cell carcinoma promote angiogenesis and endothelial gap formation in bone marrow in a time-dependent manner in a preclinical mouse model
Source: Front Oncol. 2023 Mar 23;13:1139049. doi: 10.3389/fonc.2023.1139049 (PMC10091619; doi:10.3389/fonc.2023.1139049)
Supplement: Supplementary file 1 [file DataSheet_1.pdf]

Supplementary Table 1

Category	Patients with bone metastasis (N=6)		Patients with locally advanced disease (N=6)	
Age(mean, range)	63.8(55-72)		63.5(52-76)	
Gender	Male<br>Female	5<br>1	Male<br>Female	4<br>2
Laterality	Left<br>Right	4<br>2	Left<br>Right	5<br>1
Histology	Clear cell	6	Clear cell	6
Types of Procedure	Biopsy<br>RN	2<br>4	Biopsy<br>RN	0<br>6
Clinical T stage	cT1<br>cT2<br>cT3	3<br>1<br>2	cT1<br>cT2<br>cT3	1<br>0<br>5
Clinical N stage	cN0<br>cN2	6<br>0	cN0<br>cN2	5<br>1
Metastatic site	Bone<br>Lung<br>Liver	6<br>3<br>1	None	
Pathological T stage	pT1<br>pT2<br>pT3<br>N/A	3<br>0<br>1<br>2	pT1<br>pT2<br>pT3	0<br>0<br>6
Tumor thrombus(n, %)	2(33.3%)		3(50%)	
Presurgical Treatment(n, %)	2(33.3%)		2(33.3%)	

Supplementary Figure 1

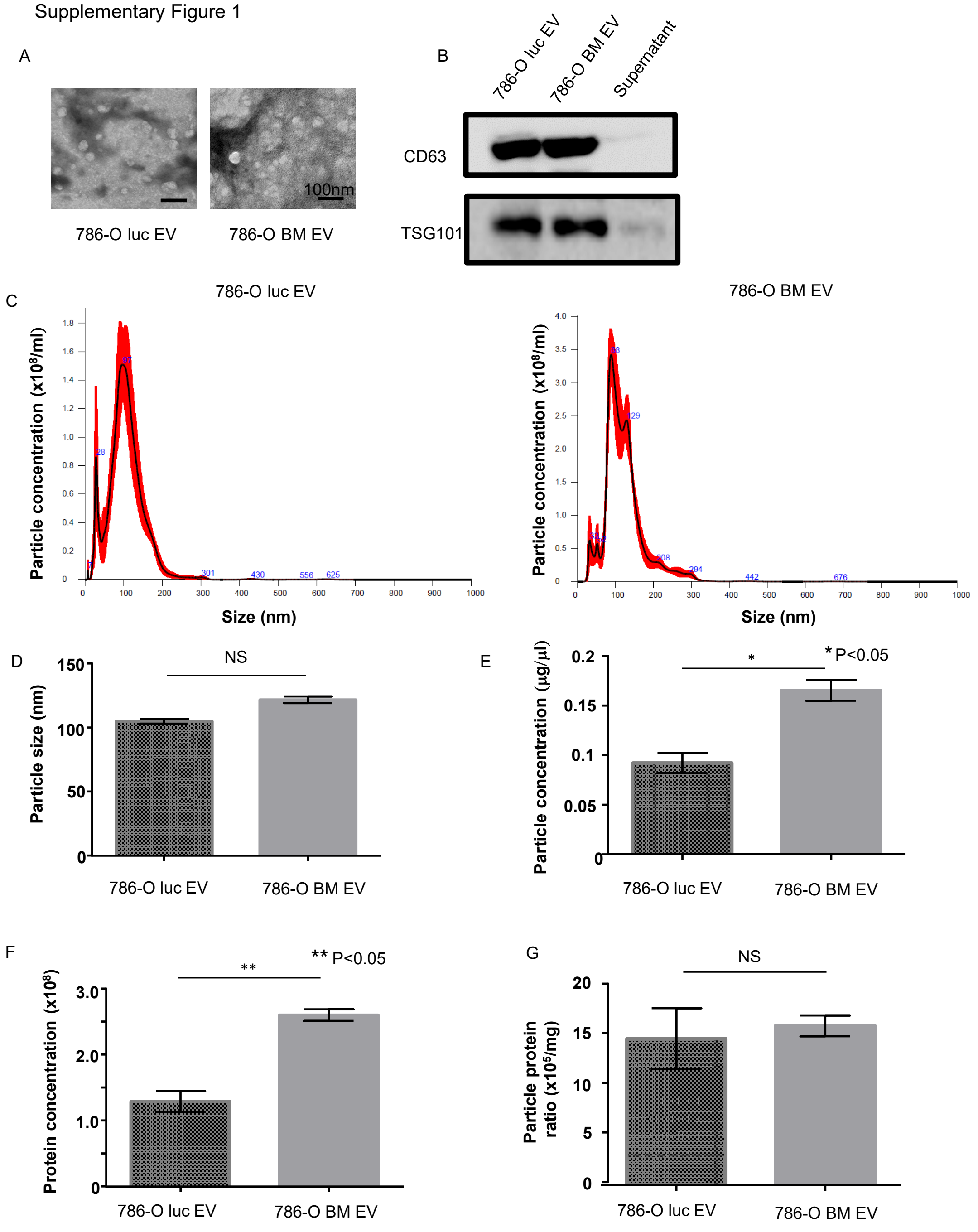

Supplementary Figure 2

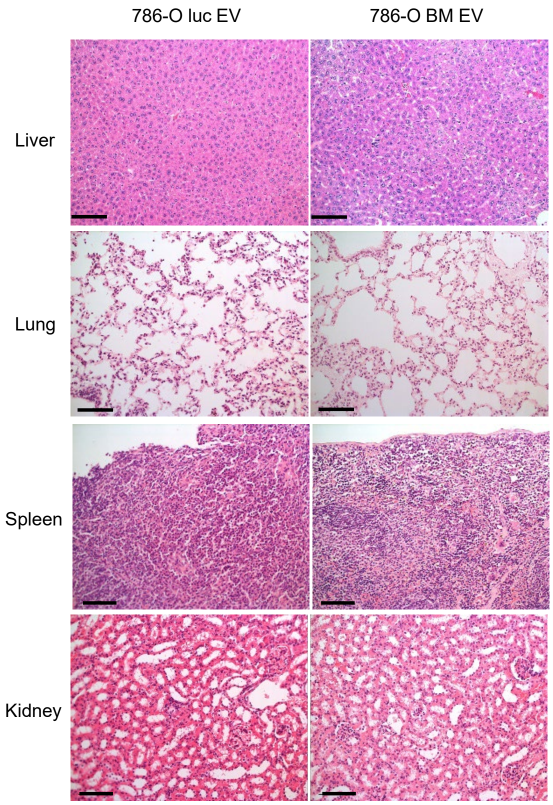

Supplementary Figure 3

A

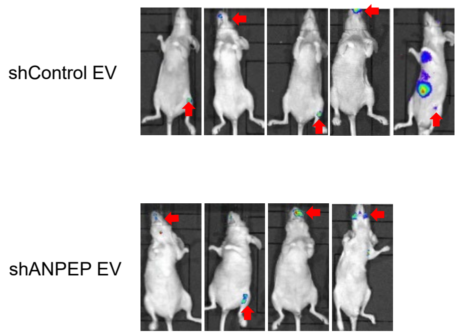

B

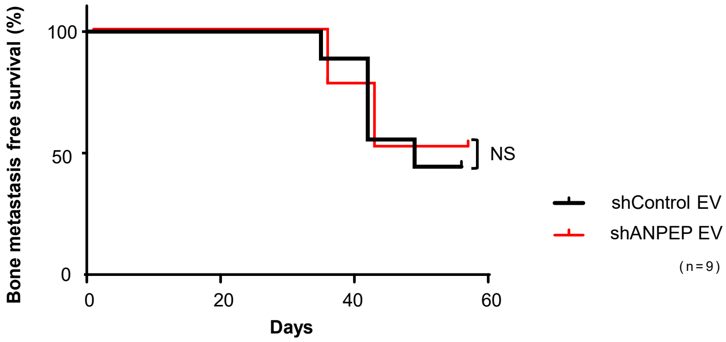

Supplementary Table 2

Description	Abundance Ratio (log2): (BM EV) / (luc EV)	Protein Localization
Gap junction alpha-1 protein OS=Homo sapiens OX=9606 GN=GJA1 PE=1 SV=2	6.64	M
Mitofusin-1 OS=Homo sapiens OX=9606 GN=MFN1 PE=1 SV=3	6.64	IC, M
Protein S100-A14 OS=Homo sapiens OX=9606 GN=S100A14 PE=1 SV=1	6.64	IC
SLIT-ROBO Rho GTPase-activating protein 2 OS=Homo sapiens OX=9606 GN=SRGAP2 PE=1 SV=3	6.64	IC
Protein S100-A8 OS=Homo sapiens OX=9606 GN=S100A8 PE=1 SV=1	5.83	IC, S
Alpha-crystallin B chain OS=Homo sapiens OX=9606 GN=CRYAB PE=1 SV=2	5.34	IC, M
Protein-arginine deiminase type-3 OS=Homo sapiens OX=9606 GN=PADI3 PE=1 SV=2	4.77	IC
Serpin B5 OS=Homo sapiens OX=9606 GN=SERPINB5 PE=1 SV=2	3.83	IC
ADP/ATP translocase 2 OS=Homo sapiens OX=9606 GN=SLC25A5 PE=1 SV=7	3.67	M
10 kDa heat shock protein, mitochondrial OS=Homo sapiens OX=9606 GN=HSPE1 PE=1 SV=2	3.47	IC
Hephaestin-like protein 1 OS=Homo sapiens OX=9606 GN=HEPHL1 PE=2 SV=2	3.24	M
Beta-hexosaminidase subunit beta OS=Homo sapiens OX=9606 GN=HEXB PE=1 SV=3	3.2	IC
Vitronectin OS=Homo sapiens OX=9606 GN=VTN PE=1 SV=1	3.13	S
Fatty acid-binding protein, adipocyte OS=Homo sapiens OX=9606 GN=FABP4 PE=1 SV=3	2.81	IC
Vesicle-associated membrane protein 3 OS=Homo sapiens OX=9606 GN=VAMP3 PE=1 SV=3	2.82	M
Aminopeptidase N OS=Homo sapiens OX=9606 GN=ANPEP PE=1 SV=4	2.54	M
Immunoglobulin heavy constant alpha 1 OS=Homo sapiens OX=9606 GN=IGHA1 PE=1 SV=2	2.29	IC
Cell surface glycoprotein MUC18 OS=Homo sapiens OX=9606 GN=MCAM PE=1 SV=2	2.24	M
Serpin B13 OS=Homo sapiens OX=9606 GN=SERPINB13 PE=1 SV=2	2.18	IC,M
Phosphate carrier protein, mitochondrial OS=Homo sapiens OX=9606 GN=SLC25A3 PE=1 SV=2	2.14	IC,M

Supplementary Table 3

Description	# Unique Peptides	Score Mascot	Score Sequest HT
Gap junction alpha-1 protein OS=Homo sapiens OX=9606 GN=GJA1 PE=1 SV=2	2	42	2.69
ADP/ATP translocase 2 OS=Homo sapiens OX=9606 GN=SLC25A5 PE=1 SV=7	1	97	8.06
Vesicle-associated membrane protein 3 OS=Homo sapiens OX=9606 GN=VAMP3 PE=1 SV=3	2	26	3.87
Aminopeptidase N OS=Homo sapiens OX=9606 GN=ANPEP PE=1 SV=4	9	301	13.97
Cell surface glycoprotein MUC18 OS=Homo sapiens OX=9606 GN=MCAM PE=1 SV=2	7	256	18.62
